# MYO18B promotes hepatocellular carcinoma progression by activating PI3K/AKT/mTOR signaling pathway

**DOI:** 10.1186/s13000-018-0763-3

**Published:** 2018-11-03

**Authors:** Zhenyu Zhang, Jinfeng Zhu, Yansong Huang, Weibing Li, Hongqiu Cheng

**Affiliations:** 1 Department of Traditional Chinese Medicine, Shenzhen Pingshan District People’s Hospital, Shenzhen, China; 2 Department of Traditional Chinese Medicine, Nantong Hospital of Traditional Chinese Medicine, Nantong, China; 3 Department of Infectious Diseases, The Second Affiliated Hospital of Shantou University Medical College, NO. 69 Dongxia North Road, Shantou, 515041 People’s Republic of China

**Keywords:** Hepatocellular carcinoma, *MYO18B*, PI3K/AKT/mTOR signaling pathway, Prognosis

## Abstract

**Background:**

*MYO18B* has been identified as a novel tumor suppressor gene in several cancers. However, its specific roles in the progression of hepatocellular carcinoma (HCC) has not been well defined.

**Methods:**

We firstly identified the expression and prognostic values of *MYO18B* in HCC using TCGA cohort and our clinical data. Then, *MYO18B* knockdown by RNA inference was implemented to investigate the effects of *MYO18B* on HCC cells. Quantitative RT-PCR and Western blot were used to determine gene and protein expression levels. CCK-8 and colony formation assays were performed to examine cell proliferation capacity. Wound healing and transwell assays were used to evaluate the migration and invasion of HepG2 cells.

**Results:**

*MYO18B* was overexpressed and correlated with poor prognosis in HCC. *MYO18B* expression was an independent risk factor for overall survival. Knockdown of *MYO18B* significantly inhibited the proliferation, migration and invasion of HepG2 cells. Meanwhile, *MYO18B* knockdown could effectively suppress the phosphorylation of PI3K, AKT, mTOR and P70S6K, suggesting that *MYO18B* might promote HCC progression by targeting PI3K/AKT/mTOR signaling pathway.

**Conclusions:**

*MYO18B* promoted tumor growth and migration via the activation of PI3K/AKT/mTOR signaling pathway. *MYO18B* might be a promising target for clinical intervention of HCC.

## Background

Hepatocellular carcinoma (HCC) is the most common primary liver cancer, representing the fifth most common cancer and the third leading cause of cancer death worldwide [1]. It is well known that persistent infections by HBV or HCV are the primary inducers of chronic liver disease, thereby resulting in liver cirrhosis and HCC [2, 3]. Aflatoxin exposure and alcohol abuse are also important risk factors for developing HCC [2]. Surveillance by biannual ultrasonography is recommended for such patients [4, 5]. Current treatment options mainly include resection, liver transplantation and interventional radiology [5]. Although recent advances in therapies have achieved improved prognoses for patients with HCC, it is still involved in a poor survival rate due to late diagnosis [6]. Currently, patients with advanced HCC lack effective therapies, representing a unique clinical challenge. Given the current burden of HCC, identifying biomarkers associated with the progression and prognosis of HCC is a promising approach to make early diagnosis, predict prognosis and develop novel therapeutic strategies.

Over the past decade, significant effort has focused on the detection of molecular alterations in HCC [7–10]. Various kinds of biomarkers (such as AFP, VEGF, hepatocyte growth factor) have been proven to harbor prognostic implications, but only serum AFP has been widely used to complement HCC surveillance and guide treatment decisions [11–13]. However, AFP levels display limitations in sensitivity and specificity [14, 15]. It is imperative to screen adequate predictive or prognostic biomarkers. *MYO18B* is a myosin family gene located at chromosome 22q12.1. Genetic instability of chromosome arm 22q has been detected in patients with HCC [16, 17], suggesting the presence of a tumor-related gene on this chromosome arm that is involved in HCC carcinogenesis. Zhu et al. [18] indicated that tumor suppressor genes on chromosome 22q11.2-22q12.1 may contribute to the pathogenesis and development of HCC. Moreover, *MYO18B* has been identified as a tumor suppressor gene whose inactivation is associated with the progression of lung cancer [19, 20], colorectal cancer [21] and ovarian cancer [22]. These observations raise the possibility that *MYO18B* is a potential cancer marker. However, the specific role of *MYO18B* in HCC progression is still unclear.

In this study, we firstly identified the expression differences of *MYO18B* between HCC tissues and healthy tissues and its prognostic value using the public data from TCGA database, and validated the results using an independent clinical cohort. Then, we investigated the specific role of *MYO18B* in HCC by experimental technique. This work purposed to reveal the significance of *MYO18B* and its underlying mechanism in the pathogenesis of HCC. The result would be of great emphasis for future control strategy of patients with HCC.

## Methods

### Patients

In this study, we identified the expression and prognostic value of *MYO18B* in HCC using two independent cohorts. The RNA-seq data of patients with HCC were obtained from TCGA data portal (https://cancergenome.nih.gov/), which contains 374 HCC samples and 50 normal samples. The other cohort contained a total of 80 patients with HCC who had undergone a resection of primary tumors at The Second Affiliated Hospital of Shantou University Medical College between 2007 and 2009. All patients were histologically confirmed as HCC. TNM classification of hepatocellular carcinoma follows 8th edition AJCC Cancer Staging system. The patients were followed up for 80 months after surgery. None of these patients received radiotherapy or chemotherapy before surgery. The clinical information of these patients was listed in Table 1. The adjacent liver tissue was obtained for control.

**Table 1:** Correlation between MYO18B expression and clinical characteristics in patients with hepatocellular carcinoma

Characteristics	Expression of MYO18B		*P* value
	Low	High	
Age			0.361
< 60	18	14	
≥ 60	22	26	
Gender			0.260
Female	20	15	
male	20	25	
Grade			1.000
G1 + G2	26	26	
G3 + G4	14	14	
Pathologic-Stage			0.025
I + II	24	14	
III + IV	16	26	
Pathologic-T			0.014
T1 + T2	26	15	
T3 + T4	14	25	
Pathologic-N			1.000
N0	37	36	
N1	3	4	
Pathologic-M			1.000
M0	39	38	
M1	1	2	

*T* tumor status, *N* regional lymph node status, *M* metastasis status

### Cell culture

Human HCC cell line HepG2 was obtained from Cell Bank of the Type Culture Collection, Chinese Academy of Sciences (Shanghai, China), and was cultured in RPMI-1640 supplemented with 10% serum, 100 U/ml penicillin, and 0.1 mg/ml streptomycin at 37 °C in a humidified incubator with 5% CO_2_.

### Transient transfection

Cells were seeded in 6-well plates at a concentration of 1 × 10^5^ per well. The next day, cells were transfected with siRNA (experimental group, si-*MYO18B*, Cat no. sc-61,119, Santa Cluz Biotechnology, Shanghai, China) or non-specific control siRNA (negative control group, si-con) using Lipofectamine 2000 (Invitrogen Life Technologies, Karlsruhe, Germany) according to the manufacture’s protocol. The transfected cells were harvested after 48-h incubation for subsequent experiments.

### Quantitative real-time PCR (qRT-PCR) assay

Total RNA was extracted from cultured cells or frozen human tissues using TRIzol Reagent (Invitrogen, Grand Island, NY, USA), and was then converted into cDNA using a PrimeScript 1st Strand cDNA Synthesis Kit (Takara, Dalian, China) according to the manufacture’s protocol. qRT-PCR assay was carried out using SYBR Green (Takara, Dalian, China). The samples were amplified as the following protocol: 95 °C for 5 min, 40 cycles of 95 °C for 30 s, 60 °C for 40 s, and 72 °C for 1 min. Primer sequences for MYO18B and GAPDH were as follows: MYO18B forward, 5’-GGAAGCAGTTAGCTGTCGC-3′ and reverse, 5’-TTGACTGGTCGTCCTGAGAGA-3; GAPDH forward, 5’-GGAGCGAGATCCCTCCAAAAT-3′, and reverse, 5’-GGCTGTTGTCATACTTCTCATGG-3′. The relative quantification was determined by 2^−ΔΔCt^ method and normalized to GAPDH. All experiments were repeated at least three times.

### Western blot

After 48-h transfection, cells were washed with PBS twice, harvested and lysed in RIPA buffer (Termo Scientifc, Rockford, IL, USA) containing protease inhibitors on ice for 30 min. The protein concentration was determined by the BCA method. Next, protein samples (20 μg) were equally loaded onto SDS-PAGE and electrotransferred to PVDF membranes. Subsequently, the membranes were blocked with 5% non-fat milk for 1 h and incubated overnight with primary antibodies (dilution 1:1000) against: MYO18B (Cat no. CBS-PA278900, Wuhan Huamei Biological Engineering Co., Ltd., Hubei, China), phosphoinositide-3 kinase (PI3K, Cat no. 4255, Cell Signaling Technology, Inc., Danvers, MA, USA), phosphorylated- (p-) PI3K (Cat no. 13857, Cell Signaling Technology, Inc.), AKT (Cat no. 9272, Cell Signaling Technology, Inc.), p-AKT (Cat no. 13038, Cell Signaling Technology, Inc.), mTOR (Cat no. 2972, Cell Signaling Technology, Inc.), p-mTOR (Cat no. 2971, Cell Signaling Technology, Inc.), p-P70S6K (Cat no. 9204, Cell Signaling Technology, Inc.), and GAPDH (Cat no. 8884, Cell Signaling Technology, Inc.). The membranes were washed with TBST buffer three times and then incubated with secondary antibodies (Anti-rabbit IgG, HRP-conjugated goat anti-rabbit, dilution 1:3000, Cat no. 7074, Cell Signaling Technology, Inc., Danvers, MA, USA) for 1 h at 25 °C. The membranes were then rinsed three times with blocking solution and visualized by the ECL detection system. The experiments were repeated at least three times for analysis. The intensity of signal on each membrane was determined using Quantity One software. GAPDH was used as a loading control for normalization of protein quantity.

### Cell proliferation assay

After transfection, cells were seeded at a density of 1000 per well in 96-well plates. Then cell viability was detected at 24 h, 48 h, 72 h and 96 h using a CCK-8 kit (Sigma-Aldrich, Merck KGaA, Darmstadt, Germany) following the manufacture’s instruction. The absorbance at 450 nm was measured using a microplate reader (Thermo Scientific Microplate Reader, Waltham, Massachusetts, USA). All assays were repeated at least three times.

### Colony formation assay

After transfection, cells were seeded at a density of 500 per well in 6-well plates and incubated in culture medium with 10% FBS for 2 weeks. During this period, the medium was replaced every 3 or 4 days. After 2 weeks, when cell colonies were formed, cells were fixed with 4% paraformaldehyde for 30 min and stained with 0.1% crystal violet (Sigma, St. Louis, MO, USA) for 30 min. Visible colonies were imaged and counted using ChemiDoc XRS image screening system (Bio-Rad). Each sample was repeated at least three times.

### Wound healing assay

Wound healing assay was used to measure cell migration capacity. Cells suspended in RPMI 1640 medium with 10% serum were seeded in 6-well plates at a density of 5 × 10^5^ cells/ml and incubated for 24 h to 100% confluence. A cell-free wound was made by scratching plates with a 100 μl plastic pipette tip. Afterwards, the cells were cultured with RPMI 1640 medium for another 24 h. The wound widths were recorded after 0 h and 24 h under an Olympus BX51 microscope (Olympus Corporation, Tokyo, Japan).

### Transwell migration and invasion assays

Transwell migration and invasion assays were performed in 24-well plates using an 8 μm pore transwell chamber (BD Biosciences, Lake Franklin, New Jersey, USA) with/without Matrigel.

In the migration assay, cells (5 × 10^3^) were seeded in the upper chamber in 100 μl serum-free RPMI 1640 medium. In the invasion assay, the transwell chambers were first coated with Matrigel, then cells (1 × 10^5^) suspended in 100 μl serum-free RPMI 1640 medium were seeded in the matrigel-coated upper chamber. The lower chambers were filled with 500 μl culture medium containing 10% serum. After incubation at 37 °C in 5% CO_2_ overnight, the wells were washed with PBS and the cells attached to the low surface were fixed with paraformaldehyde for 30 min, and stained with 0.1% crystal violet for 20 min. Cells were imaged and counted under an Olympus BX51 microscope (Olympus Corp., Shinjuku, Tokyo, Japan) over five random fields in each well. Each assay was performed in triplicate.

### Statistical analysis

Experimental values were expressed as the mean ± standard deviation, and were analyzed by SPSS Statistics software (version 22.0, Chicago, IL, USA). The data preprocessing and differential expression analysis in TCGA were performed using edgeR package [23] (version 3.20.9) in Bioconductor. The association between clinical characteristics and *MYO18B* expression was evaluated by Chi-square test. Kaplan-Meier analysis with long-rank test and Cox regression analysis were used to determine the prognostic value of *MYO18B* in HCC patients. Student’s t-test was performed to determine the significance in cell experiments. *P* < 0.05 was considered statistically significant.

## Results

### MYO18B overexpression involves poor prognosis in HCC

**Fig. 1:**
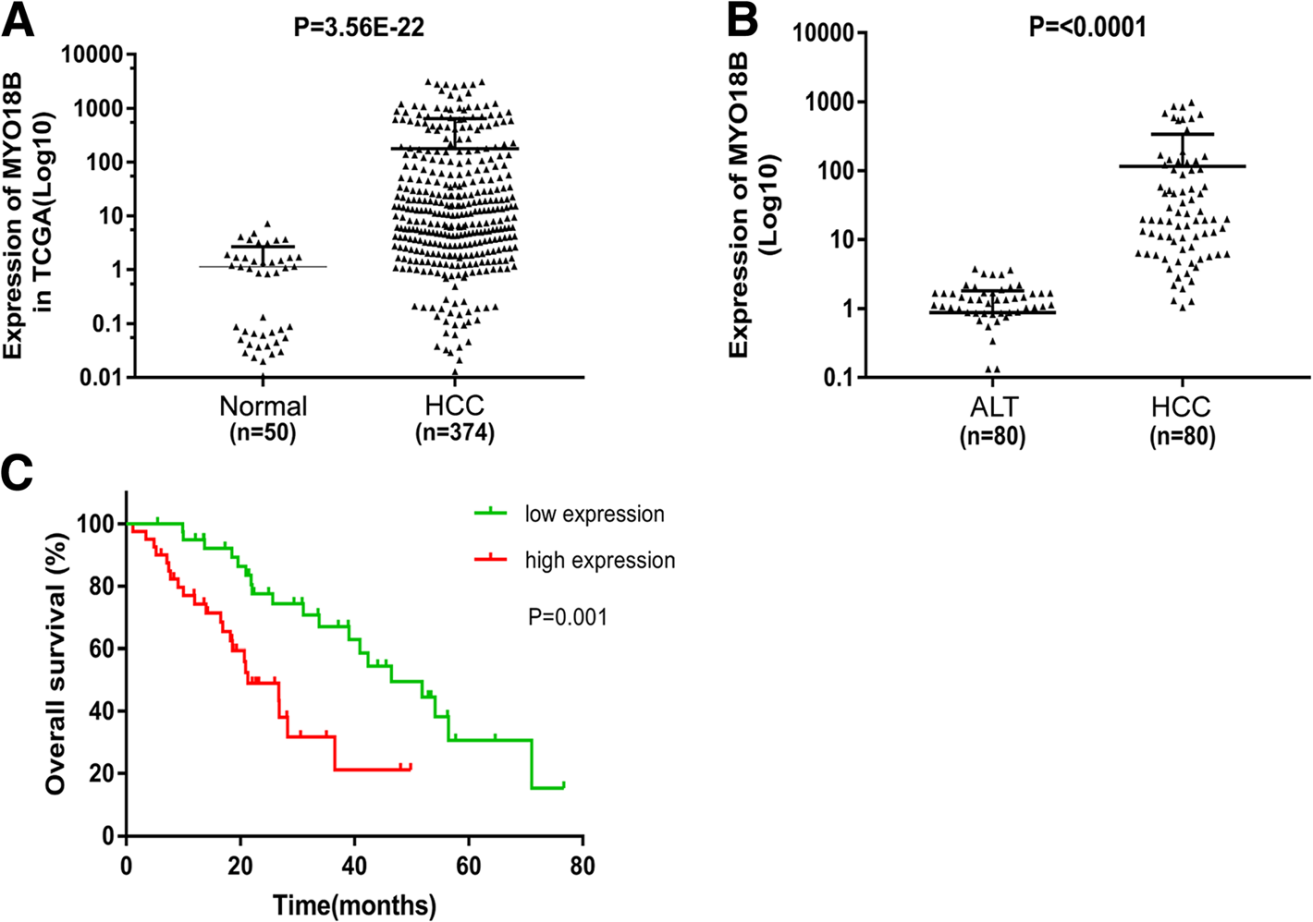
Relative MYO18B expression in tumor tissues and its clinical significance. **a** MYO18B was over-expressed in tumor tissues (*n* = 374) compared with normal tissues (*n* = 50), which was analyzed using TCGA database. **b** MYO18B was up-regulated in tumor tissues (*n* = 80) compared with adjacent liver tissues (ALT), which was analyzed by qRT-PCR. **c** Kaplan-Meier survival analysis showed that MYO18B over-expression was correlated with worse overall survival in patients with hepatocellular carcinoma (*P* = 0.001)

We first analyzed the expression differences of *MYO18B* between tumor and normal tissues using TCGA cohort. Our work found that *MYO18B* was obviously upregulated in tumor tissues when compared with normal tissues (Fig. 1a, *P* < 0.05). To validate the result from TCGA, we thus determined the expression level of *MYO18B* in 80 pairs of tumor tissues and adjacent liver tissues by qRT-PCR. The result showed that *MYO18B* expression exhibited higher levels in tumor tissues than adjacent liver tissues (Fig. 1b, *p* < 0.05). These results suggested that *MYO18B* was overexpressed in HCC tumor tissues.

To further investigate whether *MYO18B* overexpression was involved in HCC progression, we measured the correlation between *MYO18B* levels and clinical pathological characteristics in HCC patients using chi-square test. Based on the median value of *MYO18B*, 80 HCC patients were divided into high and low expression groups. As shown in Table 1, statistical analysis revealed that no significant association was found in age, gender, grade, pathologic-N and pathologic-M and *MYO18B* expression (*P* > 0.05), while *MYO18B* expression was remarkably correlated with pathologic-stage (*P* = 0.025) and pathologic-T (*P* = 0.014), implying that high expression of *MYO18B* might be involved in the progression of HCC.

Additionally, the prognostic value of *MYO18B* in HCC was evaluated by Kaplan-Meier plotting with long-rank test for difference. The result showed that HCC patients with high expression of *MYO18B* displayed a significantly poorer prognosis than those with low expression (Fig. 1c, *P* = 0.001). Cox regression analysis was further performed to analyze the prognostic value of *MYO18B* in HCC. As shown in Table 2, univariate analysis showed that *MYO18B* expression (HR = 2.993, *P* = 0.002), pathologic-stage (HR = 3.708, *p* < 0.001) and pathologic-T (HR = 3.783, *P* < 0.001) were obviously related to overall survival in HCC, while age, gender, grade, pathologic-M and pathologic-N showed no significant association with overall survival (*P* > 0.05). Multivariate analysis revealed that *MYO18B* expression was an independent prognostic factor in HCC after adjusting for other clinical variables (Table 2, HR = 2.132, *P* = 0.039). The results reveal that *MYO18B* may be a potential marker for the prognosis and progression of HCC.

**Table 2:** Cox regression analysis of overall survival in patients with hepatocellular carcinoma

Variables	Univariate analysis			Multivariate analysis		
	*P* value	HR	95% CI	*P* value	HR	95% CI
MYO18B expression (high/low)	0.002	2.993	1.505–5.952	0.039	2.132	1.038–4.380
Age(<60/≥60)	0.114	0.605	0.324–1.129			
Gender(female/male)	0.324	1.373	0.731–2.576			
Grade(G1 + G2/G3 + G4)	0.463	0.775	0.393–1.530			
Pathologic-Stage (I + II/III + IV)	0.000	3.708	1.790–7.678	0.739	1.421	0.181–11.186
Pathologic-T (T1 + T2/T3 + T4)	0.000	3.783	1.855–7.718	0.461	2.133	0.285–15.989
Pathologic-M (M0/M1)	0.052	3.267	0.991–10.769			
Pathologic-N (N0/N1)	0.080	2.351	0.902–6.130			

*HR* hazard ratio, *CI* confidence interval

### MYO18B knockdown inhibits the proliferation of HepG2 cells

**Fig. 2:**
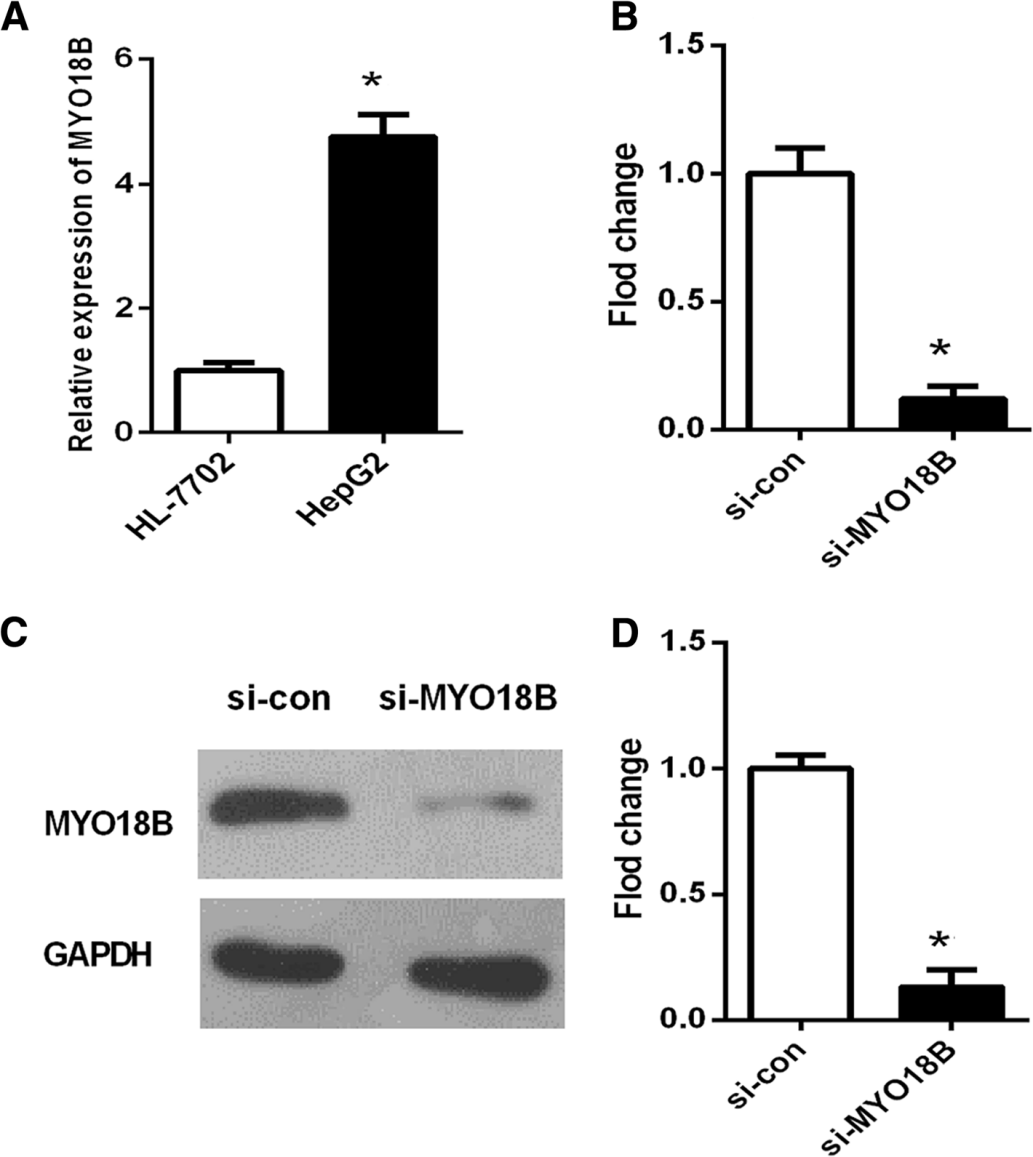
Relative MYO18B expression in hepatocellular carcinoma cells. **a** The expression levels of MYO18B in HepG2 and HL-7702 cell lines were analyzed by qRT-PCR. MYO18B expression displayed a higher level in HepG2 cells. **b**-**d** HepG2 cells were transfected with one specific siRNA (experimental group, si-MYO18B) or non-specific control siRNA (negative control group, si-con). MYO18B expression was determined by qRT-PCR (**b**) and western blot (**c**, **d**) respectively. *P < 0.05 versus si-con group

We detected the mRNA expression of *MYO18B* in HCC cell line HepG2. Human normal liver cells HL-7702 were used as a control. qRT-PCR analysis found that *MYO18B* expression was significantly upregulated in HepG2 cells than that in HL-7702 cells (Fig. 2a, *p* < 0.05).

To investigate the effects of *MYO18B* on HCC, HepG2 cells were transfected with *MYO18B* siRNA or control siRNA, respectively. qRT-PCR and Western blot were utilized to determine the mRNA and protein levels of *MYO18B*, respectively. The result indicated that *MYO18B* expression was significantly downregulated by *MYO18B* siRNA in both mRNA (Fig. 2b, *P* < 0.05) and protein levels (Fig. 2c and d, *P* < 0.05).

**Fig. 3:**
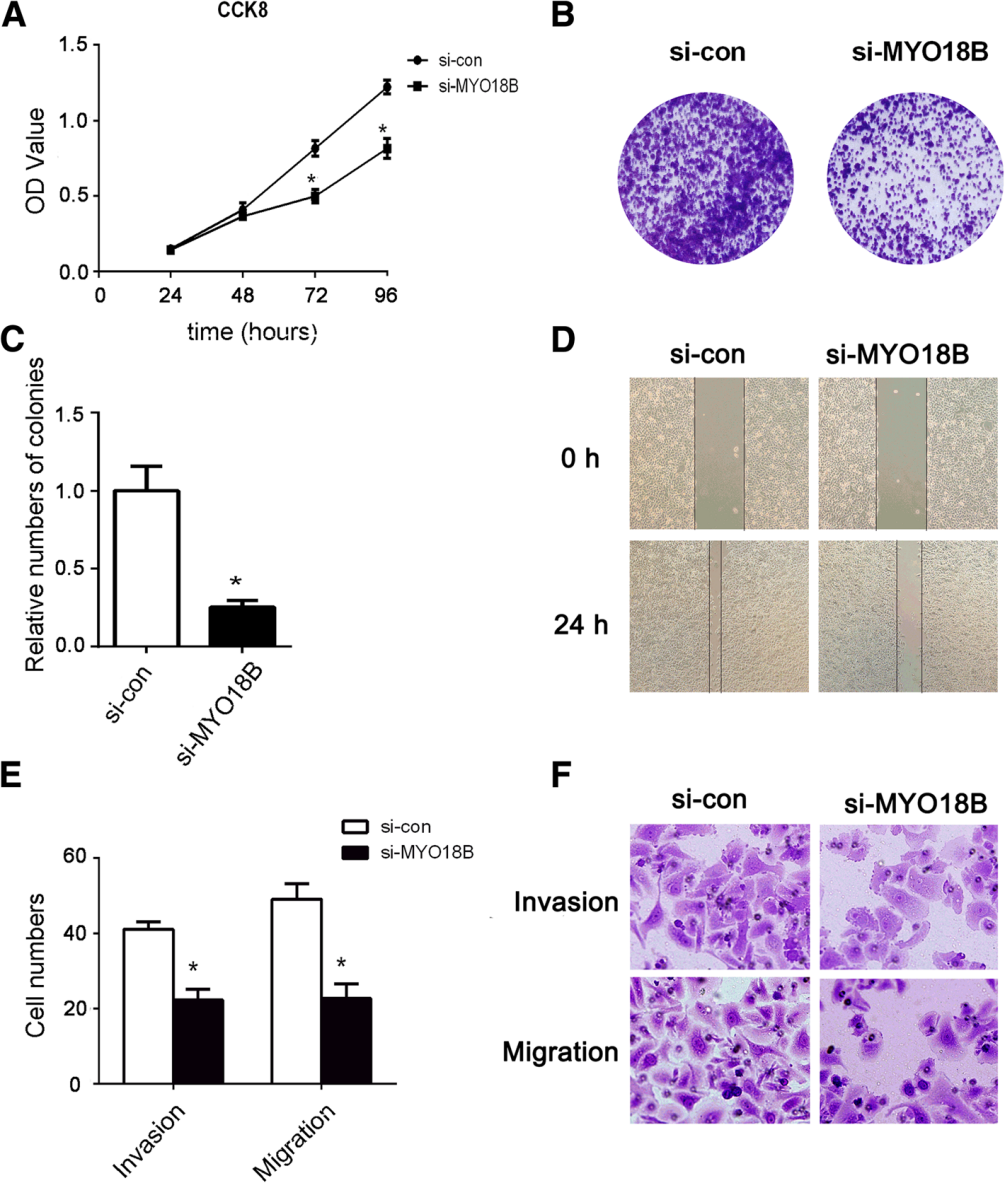
Knockdown of MYO18B inhibited the proliferation, migration and invasion of HepG2 cells. **a** CCK-8 assays were performed to measure the proliferation of the transfected HepG2 cells, which showed knockdown of MYO18B (si-MYO18B group) obviously inhibited the cell growth. **b**, **c** Colony formation assays were used to determined the proliferation of the transfected HepG2 cells, which also revealed knockdown of MYO18B (si-MYO18B group) obviously inhibited the cell growth. **d** After incubated the transfected cells for 24 h, knockdown of MYO18B (si-MYO18B group) significantly suppressed the migration of HepG2 cells analyzed by wound healing assays. **e**, **f** Transwell assays further demonstrated that knockdown of MYO18B (si-MYO18B group) obviously inhibited the migration and invasion of the transfected HepG2 cells. *P < 0.05 versus si-con group

To study the effect of *MYO18B* on cell proliferation, the viability of HepG2 cells was detected by CCK-8 and colony formation assays. As shown in Fig. 3a, CCK-8 assay revealed that *MYO18B* knockdown significantly suppressed the proliferation of HepG2 cells after 72 h and 96 h of transfection when compared to control group (*P* < 0.05). In addition, Fig. 3b showed the representative images of the size and number of colonies per well. Colony formation assay showed that *MYO18B* knockdown significantly decreased the number of colony formation (Fig. 3c *P* < 0.05).

### MYO18B knockdown inhibits the migration and invasion of HepG2 cells

HCC cells are characterized by high migration ability. To evaluate the effect of *MYO18B* expression on the cell migration and invasion, wound healing and transwell assays were carried out. As shown in Fig. 3d, cell culture images were captured at 0 and 24 h and the wound gap was analyzed. The wound healing assay showed that the wound closure in si-*MYO18B* group was obviously slower compared with the control group, indicating that *MYO18B* knockdown suppressed the migration activity of HepG2 cells. Transwell results showed that the number of the invading and migrating cells in the si-*MYO18B* group was significantly decreased than that in the control group (Fig. 3e and f, *P* < 0.05), suggesting that *MYO18B* knockdown attenuated the migration and invasion of HepG2 cells.

### Knockdown of MYO18B inhibited the activation of PI3K/AKT/mTOR pathway

**Fig. 4:**
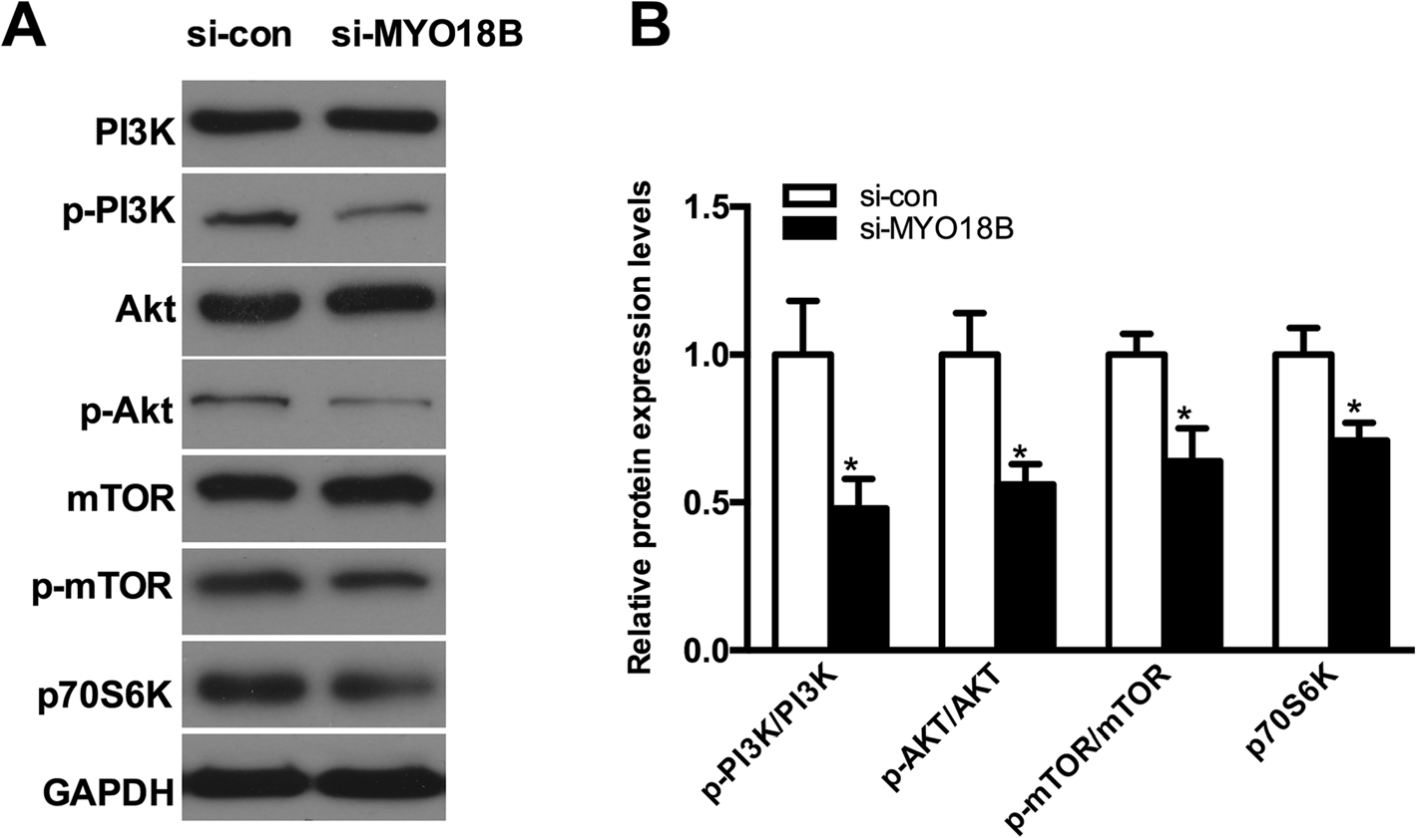
The effect of MYO18B on PI3K/AKT pathway. **a** Western blot assays were used to measure the expression of PI3K, p-PI3K, AKT, P-AKT, mTOR, p-mTOR and p-P70S6K proteins in the transfected HepG2 cells. **b** Quantification of PI3K, p-PI3K, AKT, P-AKT, mTOR, p-mTOR and p-P70S6K proteins was performed and normalized to the GAPDH a loading control. *P < 0.05 versus si-con group

PI3K/AKT/mTOR signaling pathway plays important roles in regulating multiple cellular functions, such as cell proliferation, differentiation and intracellular trafficking [24]. To explore the underlying mechanism of *MYO18B* in HCC, we analyzed the protein levels of PI3K/AKT/mTOR signaling pathway in HepG2 cells by Western blot. As shown in Fig. 4, the protein levels of PI3K, AKT and mTOR had no significant differences between si-*MYO18B* group and control group, but *MYO18B* knockdown significantly suppressed the phosphorylation of PI3K, AKT and mTOR, and led to an obvious decrease of p70S6K expression (*P* < 0.05). The results suggested that *MYO18B* knockdown might suppress the proliferation, migration and invasion of HepG2 cells by inhibiting the activation of PI3K/AKT/mTOR signaling pathway.

## Discussion

In this study, we investigated the expression and prognostic values of *MYO18B* in HCC using two independent cohorts, and found that *MYO18B* was overexpressed in HCC, and *MYO18B* high expression was involved in a poor prognosis in HCC. Silencing of *MYO18B* in HepG2 cells could inhibit cell proliferation, migration and invasion via attenuating the activation of PI3K/AKT/mTOR signaling pathway. Therefore, targeting *MYO18B* might represent a novel adjuvant clinical intervention for patients with HCC.

MYO18B is a novel unconventional myosin heavy chain that mainly expresses in human cardiac and skeletal muscles [25]. It is associated with the ATPase proteasome subunit Sug1 and is a substrate for proteasomal degradation [26]. Previous studies identified *MYO18B* as a tumor suppressor gene in several cancers [19–22]. Nishioka et al. [20] illustrated that *MYO18B* expression was decreased in 88% of nonsmall cell lung carcinoma and 47% of small cell lung carcinoma cell lines, and restoration of *MYO18B* expression can effectively suppress the growth of lung cancer cells. Nakano et al. [21] showed that 82% colorectal cancer cell lines presented reduced *MYO18B* expression, which was restored in all 9 by treatment. Yanaihara et al. [22] indicated that *MYO18B* expression was reduced in all 4 ovarian cancer cell lines and in 71% of primary ovarian cancers. Yokota et al. [27] demonstrated that *MYO18B* was involved in tumor suppression by regulating cell movement and maintaining cell structure. Nobutaka et al. [28] found that none of human malignant pleural mesothelioma cell lines expressed a detectable level of *MYO18B*, and a restored expression of *MYO18B* inhibited cell growth and increased cell apoptosis. Until now, *MYO18B* remains a mysterious player in HCC. Thus we attempted to investigate the specific effects of *MYO18B* on the progression of HCC. In the present study, we found that *MYO18B* expression was significantly upregulated in both HCC tumor tissues and HCC cell line, and high level of *MYO18B* was correlated with a poor prognosis for patients with HCC. Furthermore, *MYO18B* expression was an independent prognostic factor in HCC. In addition, experimental analysis showed that *MYO18B* promoted the proliferation, migration and invasion of HCC cells. Evidently, our results were inconsistent with the previous findings that *MYO18B* was a tumor suppressor gene and downregulated in cancer tissues. The occurrence of opposite results might partially due to the tissue-specific expression of *MYO18B*. Moreover, previous studies gave the conclusion based a relative small sample size. While, our finding was drawn using two large independent cohorts, suggesting a more reliable result. Even though, further studies should be performed to elucidate the true circumstance behind the inconsistence.

To explore the underlying mechanism of *MYO18B* in HCC, we evaluated the effect of *MYO18B* on the PI3K/AKT/mTOR signaling pathway. PI3K/AKT/mTOR signaling pathway is a major activated signaling pathway in human malignancies, and regulates multiple cellular processes, such as cell proliferation, apoptosis and migration [29–31]. The PI3K/Akt pathway is a key regulator of cell survival via inhibition of apoptosis in various types of human cancers [32–34]. Akt activation promotes metastasis and invasion of cancer cells, and phosphorylates mammalian target of rapamycin (mTOR) [35]. mTOR is an important downstream target of PI3K/Akt, and positively regulates the serine/threonine kinase p70 S6 kinase (p70S6K) [35]. Previous studies demonstrated frequent changes of the PI3K/Akt/mTOR pathway in HCC [24, 36, 37]. Components of this pathway are frequently deregulated in an extensive number of tumors, making PI3K/AKT/mTOR signaling pathway an attractive target for cancer therapy [30, 38, 39]. Increasing evidence indicates that inhibition of PI3K/Akt/mTOR pathway suppresses cell growth in many tumor types [24, 40–42]. In this report, we found that the phosphorylation levels of PI3K, Akt and mTOR were significantly suppressed by *MYO18B* knockdown in HepG2 cells, suggesting that *MYO18B* knockdown could inhibit the activation of PI3K/AKT/mTOR signaling pathway in HCC.

We acknowledge some limitations of our study. In particular, we determined the expression of MYO18B in HCC tumor tissues using two independent cohorts, one from TCGA and one from our clinical patients. In TCGA, the normal liver tissues were used for control, whereas the adjacent liver tissues were used for control in our clinical cohort. Most of HCCs develop in cirrhotic livers, therefore the tumor adjacent tissue is mostly not normal. A further limitation is that some clinical characteristics of these 80 HCC patients such as tumor size, tumor number, and lymphovascular invasion, background liver condition were not included, because the information was not available for a substantial proportion of cases. Thus, the significance and robustness of MYO18B as a biomarker requires further confirmation.

## Conclusions

*MYO18B* was overexpressed in HCC and correlated with poor prognosis in HCC. *MYO18B* expression was an independent prognostic factor in HCC. *MYO18B* knockdown suppressed cell proliferation, migration and invasion via inhibiting the activation of PI3K/AKT/mTOR signaling pathway. Targeting *MYO18B* might represent a promising target for HCC treatment.

## Abbreviations

HCC: Hepatocellular carcinoma
qRT-PCR: Quantitative real-time PCR
TCGA: The Cancer Genome Atlas
